# A randomized double-blind controlled trial of the use of dydrogesterone in women with threatened miscarriage in the first trimester: study protocol for a randomized controlled trial

**DOI:** 10.1186/s13063-016-1509-8

**Published:** 2016-08-17

**Authors:** Diana Man Ka Chan, Ka Wang Cheung, Sofie Shuk Fei Yung, Vivian Chi Yan Lee, Raymond Hang Wun Li, Ernest Hung Yu Ng

**Affiliations:** Department of Obstetrics and Gynaecology, Queen Mary Hospital, 102 Pokfulam Road, Hong Kong Special Administrative Region, China

**Keywords:** Threatened miscarriage, Progestogen, Dydrogesterone, Randomized controlled trial

## Abstract

**Background:**

Miscarriage is a common complication of pregnancy occurring in 15–20 % of all clinically recognized pregnancies. Currently, there is still no good scientific evidence to support the routine use of progestogens for the treatment of threatened miscarriage because the existing studies were not large enough to show a significant difference and some of them were not randomized or double-blind.

**Methods:**

This is a double-blind, randomized controlled trial. A total of 400 patients presenting with first-trimester threatened miscarriage will be enrolled. They will be randomized to take dydrogesterone 40 mg per os, followed by 10 mg per os three times a day or placebo until twelve completed weeks of gestation or 1 week after the bleeding has stopped, whichever is longer. The primary outcome is the percentage of miscarriage before 20 weeks of gestation.

**Discussion:**

We postulate that the dydrogesterone therapy will significantly reduce the risk of miscarriage in women with threatened miscarriage.

**Trial registration:**

This study is registered at ClinicalTrials.gov, NCT02128685. Registered on 29 April 2014.

**Electronic supplementary material:**

The online version of this article (doi:10.1186/s13063-016-1509-8) contains supplementary material, which is available to authorized users.

## Background

Miscarriage is a common complication of pregnancy occurring in 15–20 % of all clinically recognized pregnancies [1] and it may be associated with significant physical and psychological sequelae. Threatened miscarriage is manifested by vaginal bleeding, with or without abdominal pain, while the cervix is closed and the fetus remains viable inside the uterine cavity.

The reasons of miscarriages are many. During the first trimester, the most common cause of miscarriage is embryonic chromosomal abnormality [2], although in some cases the cause cannot be identified. Progesterone plays a crucial role in the maintenance of pregnancy. It is secreted by the corpus luteum that provides early pregnancy support until placental production takes over at 10 to 12 weeks of gestation. Low levels of circulating progesterone have been linked to impending miscarriage [3]. It has been postulated, therefore, that a lack of progesterone is a cause of miscarriage rather than a secondary signal of failing pregnancy.

Treatment options for threatened miscarriage include bed rest, the use of human chorionic gonadotropin (hCG), uterine muscle relaxants and progestogens. Bed rest is conventionally the most commonly used management technique for threatened miscarriage. However, there is little evidence of its value. A recent Cochrane review also came to the conclusion that there is insufficient evidence to support a policy of bed rest to prevent miscarriage [4]. Indeed, a lack of physical activity can lead to other complications such as thromboembolic events, back pain and muscle atrophy, and women may experience emotional, family related and economic stress during bed rest, as well as self-blame if they fail to comply and subsequently suffer a miscarriage.

A small randomized study conducted by Harrison [5] showed hCG to be significantly more effective than bed rest. Based on this finding, a double-blind, randomized, placebo-controlled study was conducted in 183 women with first-trimester vaginal bleeding and a viable fetus as confirmed by ultrasound [6]. However, there was no significant difference in the incidence of complete miscarriage between the hCG and placebo groups (12 % versus 11 %). The sample size of the study was smaller than planned due to loss of patients in follow-up and it was possible that the sample size was too small to reveal any differences in effect between hCG and placebo. Uterine muscle relaxing drugs, which include beta-agonists and atropine-like antispasmodic agents, are rarely used today. A recent search of the Cochrane Pregnancy and Childbirth Group Trials register and Central Register of Controlled Trials confirmed that there is insufficient evidence to support their use [7].

Progestogens have been used to treat threatened miscarriage for many years. In 1989, Goldstein et al. published a meta-analysis of 15 randomized controlled trials and concluded that progesterone and its analogs are ineffective in the maintenance of pregnancy in women with threatened miscarriage [8]. Some recent studies suggest that the use of progestogen is associated with reduction in risk of miscarriage in women with threatened miscarriage [9–11]. It has been suggested that progesterone potentially sustains the survival of the embryo by shifting the immune system towards production of non-inflammatory T-helper 2 cytokines and by increasing nitric oxide production, thus improving blood flow and oxygen supply [12, 13]. Progesterone also plays an important role in keeping the myometrium quiescent, as demonstrated by enhanced uterine contractility and sensitivity to prostaglandins, after treatment with mifepristone, an anti-progestogen [14].

A recent Cochrane review assessing the efficacy and safety of progestogens in threatened miscarriage identified four trials to be included in a meta-analysis, involving 421 participants, which compared progesterone with either placebo or no medication [15]. It concluded that progestogen treatment for threatened miscarriage reduced the risk of miscarriage by 47 % with a confidence interval consistent with a risk reduction of 21 to 65 %. Only in a subgroup of women who were treated with vaginal progesterone was the treatment not statistically effective in reducing miscarriage. It also showed no statistically significant difference in the number of congenital abnormalities, pregnancy-induced hypertension nor antepartum hemorrhage between the women who received progestogens and those who did not. However, it was commented that the results of this systematic review should be approached with caution due to the poor methodological quality of some of the included trials, which were not double-blind, and the small number of participants.

Dydrogesterone, a retro progesterone with very good oral bioavailability, is structurally and pharmacologically very similar to natural progesterone. It is considered suitable for women with threatened miscarriage as, in contrast to other available synthetic progestogens, it does not have androgenic side effects in the mother (e.g., hirsutism, acne) or estrogenic effects on the fetus [11]. It also does not alter the normal secretory transformation of the endometrium, nor inhibit the formation of progesterone in the placenta [10]. Moreover, studies have shown that it increases the proportion of progesterone-induced blocking factor-positive cells, while stimulating the production of T-helper 2 cytokines and inhibiting T-helper 1 cytokines [16–20], thereby ensuring protective immunomodulation. Regarding its safety in pregnancy, despite some early suggestions that progestogens may increase the risk of congenital developmental disorders [21, 22], evidence from subsequent large prospective studies and meta-analyses indicates that any such teratogenic effects are unlikely [23–25]. A recent review of maternal use of dydrogesterone during pregnancy also found no evidence for an increased risk of congenital malformations [26].

Hence, there is still no good scientific evidence to support the routine use of progestogens for the treatment of threatened miscarriage because the existing studies were not large enough and some of them were not randomized or double-blind. Therefore, a large randomized double-blind trial is needed.

## Objective and hypothesis

The aim of this study is to determine whether dydrogesterone therapy reduces miscarriage in women with first-trimester threatened miscarriage. The hypothesis is that dydrogesterone therapy will significantly reduce the risk of miscarriage in this group of women.

## Methods

### Trial design (Table 1)

This is a double-blind, randomized controlled trial. The study will be conducted in two public hospitals in Hong Kong: Queen Mary Hospital and Kwong Wah Hospital. Queen Mary Hospital is the affiliated hospital of the Faculty of Medicine, the University of Hong Kong. An ethics approval has been obtained from the Institutional Review Board of the University of Hong Kong/Hospital Authority Hong Kong West Cluster (Ref number: UW 13-292). Written informed consent will be sought from subjects at the time of recruitment, including consents for collection of tissue mass and for further chromosomal and molecular studies. The clinical trial protocol also follows the SPIRIT 2013 checklist (Additional file 1).

**Table 1:** The schedule of enrollment, interventions and assessments^a^

	Study period					
	Enrollment	Allocation	Post-allocation			Close-out
Timepoint^b^	3/2016 until present	0	Weekly until 12 completed gestational weeks or 1 week after bleeding has stopped, whichever is longer	20 weeks	Until delivery	24th month
Enrollment:						
Eligibility screen	X					
Informed consent	X					
(List other procedures)						
Allocation		X				
Interventions:						
(Dydrogesterone)			X			
(Placebo)			X			
(List other study groups)						
Assessments:						
Pelvic scan, serum hCG and progesterone			X			
(Primary outcome)				X		
(Secondary outcomes)					X	X

^a^Recommended content can be displayed using various schematic formats. See *SPIRIT 2013 Explanation and Elaboration* for examples from protocols [27]

^b^List of specific timepoints in this row

*hCG* human chorionic gonadotropin

### Selection and withdrawal of subjects

The population will be women presenting with threatened miscarriage between five and twelve completed weeks’ gestation. Threatened miscarriage is defined as vaginal bleeding, with or without abdominal pain, in a pregnant woman with pelvic ultrasound confirming an intrauterine gestational sac or fetus(es) with positive fetal heart pulsation.

#### Inclusion criteria

- Age of women from 18 to 40 years at the time of recruitment (not beyond 40th birthday)
- Absence of fever (temperature ≥38.5 °C)
- Gestation less than twelve completed weeks as defined by pelvic ultrasound
- Presence of intrauterine gestational sac(s) if a urinary pregnancy test is first positive within the past 2 weeks
- Presence of intrauterine fetus(es) with positive fetal heart pulsation or presence of intrauterine fetus(es) with crown-rump length of less than 7 mm and no fetal pulsation on pelvic scanning

#### Exclusion criteria

- Age of women older than 40 years at the time of recruitment
- History of recurrent miscarriage defined as three or more consecutive spontaneous miscarriages
- History of known parental chromosomal abnormalities
- Heavy vaginal bleeding requiring surgical intervention
- Severe abdominal pain requiring surgical intervention
- Absence of cardiac pulsation in a fetal pole with crown-rump length of ≥7 mm on transvaginal scanning
- Use of hCG or progesterone treatment for threatened miscarriage prior to recruitment
- Women with current or suspected breast or genital cancers, hepatic disease or tumors

### Treatment of subjects

Subjects who consent to participate will undergo the following procedures:

1. Background history*: age, race, medical history, obstetric and gynecological history, last menstrual period, estimated gestational age at entry
2. Clinical history and findings*: severity of symptoms and ultrasound findings
3. Physical examination*
4. Blood for serum hCG and progesterone levels
5. Treatment: dydrogesterone (treatment group) or placebo (control group)

*Procedures should be undertaken upon admission or in the Early Pregnancy Assessment Clinic

By computer-generated randomization, patients will be assigned into one of the following two groups: the progesterone and control groups. The randomization process will be done using the computer in a 1:1 ratio in a block of 10. Each randomization result will be put into a sealed opaque envelop. One envelop will be opened if a woman agrees to join the study. Both the clinicians and patients will be blinded from the treatment given. An unblinding procedure will be considered if there are adverse drug reactions after treatment as deemed necessary by the clinician in charge.

Patients allocated to the dydrogesterone group will receive dydrogesterone 40 mg per os, followed by 10 mg per os three times a day, and an identical-looking placebo will be used in the control group accordingly. Concomitant use of any other hormonal medications or tocolytic agents will not be allowed. Patients will be followed up with weekly pelvic ultrasound until twelve completed weeks of gestation or 1 week after the bleeding has stopped, whichever is longer. Blood will be taken weekly for serum hCG and progesterone levels. The treatment will be continued until twelve completed weeks of gestation or 1 week after the bleeding has stopped, whichever is longer. Any adverse effects from drugs and the patient’s compliance will be noted during follow-up. For serious and unexpected adverse drug reactions, a report on the adverse drug reaction will be submitted to the Drug Office, Department of Health as soon as possible but no later than seven calendar days after first becoming known to the investigators. Non-serious adverse reactions and expected serious adverse reactions will be reported in a brief summary at the conclusion of the trial. Patients will receive a standard antenatal checkup and follow-up routinely in the antenatal clinic until delivery. Written consent regarding retrieval of pregnancy and delivery data will be sought from the patient at the time of study entry. The obstetric outcomes will be traced from the electronic patient record system if the patients deliver in public hospitals. A pre-formatted letter with replying address available will be given to the patient at the end of the study period and is to be filled by the private obstetrician and returned to us after delivery. If no reply letter is received 2–3 months after the expected date of confinement of the patient, a letter including the patient’s authorization will be sent to the corresponding private obstetrician to retrieve the information of the pregnancy outcomes.

Treatment will be stopped if the vaginal bleeding becomes severe and requires surgical intervention, or a diagnosis of silent miscarriage is confirmed upon a follow-up scan (i.e., the gestational sac or fetal pole fails to grow after 1 week, or there is no cardiac activity in a fetal pole with crown-rump length of ≥7 mm). If the patient has a spontaneous miscarriage, the tissue mass passed or obtained after medical or surgical evacuation will be sent for histology and karyotyping by quantitative fluorescence-polymerase chain reaction (QF‐PCR) or the array comparative genomic hybridization method. QF-PCR, which is a simple and cheap method, will first be used to exclude common aneuploidy of chromosomes 13, 18, 21 and XY. The array comparative genomic hybridization method will be employed in those with negative QF-PCR results to confirm or exclude aneuploidy.

The primary outcome is the percentage of miscarriages before 20 weeks of gestation. The secondary outcomes are the live birth rate; the proportion of heavy vaginal bleeding or severe abdominal pain requiring surgical intervention (after 20 weeks); and obstetric complications including antepartum hemorrhage, placenta previa, pregnancy-induced hypertension, intrauterine death, congenital abnormality, preterm labor, and low birth weight at term. The birth weight, gestational age at delivery and apgar score are also included. The definitions of the obstetric complications are as follows:

- Antepartum hemorrhage: any vaginal bleeding during pregnancy from the 24th week of gestational age to term
- Placenta previa: placenta inserting partially or wholly in the lower uterine segment, diagnosed by antenatal ultrasound at the second and third trimesters
- Pregnancy-induced hypertension: development of new-onset hypertension (blood pressure persistently 140/90 mmHg or higher on two occasions at least 4 hours apart) during pregnancy after 20 weeks’ gestation, labor or the puerperium in a previously normotensive non-proteinuric women
- Intrauterine death: fetal death in utero after 24 weeks’ gestation
- Preterm labor: any premature spontaneous delivery from 24 weeks’ to 36 weeks’ gestation
- Low birth weight at term: baby born with birth weight less than 2500 g at or after 37 weeks’ gestation

In order to increase the generalizability of our results, we do not exclude subjects with multiple pregnancy and uterine anomaly, and we also include pregnancy of uncertain viability. However, this decision may potentially increase the heterogenicity of the results and this may be one of the limitations of the study.

### Statistics

#### Statistical tests

All statistical analyses will be performed using IBM SPSS software using intention-to-treat and per protocol analyses. Nominal data will be described by frequencies and percentages, and correlations will be analyzed using the chi-square test; continuous data will be expressed as mean ± standard deviation or median (range), and analyzed using Student’s *t* test or the Mann-Whitney *U* test depending on the normality of the data. The primary outcome, the percentage of miscarriage before 20 weeks of gestation, will be analyzed with the chi-square test. Subgroup analysis for the primary outcome will be done with regard to gravida, gestation at presentation, presence of subchorionic hematoma and after exclusion of those with an abnormal karotype in the abortus. The secondary outcomes will be compared between the treatment group and the control group by the chi-square test. Logistic regression analysis will be performed to find out the independent predictive factors for miscarriage. A *p* value <0.05 will be considered as statistically significant.

Every attempt will be made to collect full follow-up data on all women (unless a woman withdraws consent for follow-up data collection). In particular, women will be followed up even after protocol violation. It is thus anticipated that missing data will be minimal. Women with missing primary outcome data will not be included in the primary analysis. This presents a risk of bias, and secondary sensitivity analyses will be undertaken to assess the possible impact of the risk, including the assumption that all women lost to follow-up have miscarriages before 20 weeks. Other sensitivity analyses will involve simulating missing responses using a multiple imputation.

#### Sample size estimation

Based on the two previous studies [11, 12], with the pooled miscarriage rate in the progestogen group and control group being 27/182 (14.8 %) versus 42/155 (27.1 %), respectively, a sample size of 171 per group is needed to achieve a power of 80 % with a type I error of 0.05. To allow for drop out, we plan to recruit 400 subjects in total with 200 patients in each arm.

## Discussion

The control group will only receive placebo in this study. Currently, there is still no good scientific evidence to support the routine use of progestogens for the treatment of threatened miscarriage because the existing studies were not large enough to show a significant difference and some of them were not randomized or double-blind. Currently, we are not offering any drug treatment in public hospitals in Hong Kong for patients presenting with threatened miscarriage, which is the way that subjects in the control group will be managed. Hence, there is no ethical concern in this regard.

In order to increase the generalizability of our results, we do not exclude subjects with multiple pregnancies and uterine anomaly, and we also include pregnancy of uncertain viability. However, this decision may potentially increase the heterogenicity of the results and this may be one of the limitations of the study.

### Trial status

The study was started in March 2016. An ethics approval has been obtained from the Institutional Review Board of the University of Hong Kong/Hospital Authority Hong Kong West Cluster (Ref number: UW 13-292).

## Abbreviations

hCG, human chorionic gonadotropin; QF-PCR, quantitative fluorescence-polymerase chain reaction

## Additional file

Additional file 1: SPIRIT fillable-checklist-miscarriage. (DOC 97 kb)
